# Monthly Summary

**Published:** 1890-06

**Authors:** 


### Monthly Summary.

DR. B. H. CATCHING, of Atlanta, Ga., is at work on a Compendium of Practical Dentistry, which will consist mainly of the valuable matter contained in the various dental journals for the year 1890. This compendium will be a ready-reference book—a sort of annual encyclopedia of practical dentistry, and it is proposed to issue a volume each year. The price is fixed at $2.50, to be paid on delivery of book.

A DENTAL OPERATION IN WHICH TEETH BURIED IN THE JAW ARE REMOVED AND PAIN ALLEVIATED.—Anson Washburn, the fourteen-year-old son of Austin Washburn, of the Bee Line, sat in Dr. J. B. Morrison's office yesterday reading a paper and fanning himself unconcernedly. He has passed through one of the most remarkable operations known in dental surgery. When he was about five years of age he had an attack of scarlet fever that caused the retention of four teeth on the right side of the upper jaw. Dr. Morrison made an exploration and found the teeth and drew two of them down. One of them did not require much attention, except careful watching. When exploring for the eye tooth, he found it between the hard palette and the floor of the nose, pointing toward the left jaw. The tooth was imbedded in a sack of pus and the most careful treatment was needed in removing the pus and to prevent it from aggravating the cut parts and causing blood poison. The tooth and its bony attachments were cut loose, carefully cleaned of all foreign substances, and placed in its proper position. The central incisor was kept out of the boy's mouth for two and a half hours. The teeth that were changed about are growing nicely, and young Washburn said that he is suffering no pain, no inflammation has set in, and his teeth are in good condition. He was thoroughly under the influence of ether during the operation. The operation is out of the ordinary.—"Indianapolis News," June 25, '90.

---

OUR POSITION AT THE CHAIR.—Our position at the chair is not of small moment. By the habits of some dentists, one would suppose the patient has no rights the dentist is bound to respect. He lolls and leans and crowds on his patient, making of him his cushion, his staff and his main support, till the patient feels crushed and smothered and exhausted. We have seen a dentist so rudely lean and press against his lady patients as to be repulsive and immodest. There may be no resistance in plain English to all this, "but it is remembered" and "reported," much to the dentist's detriment.

Then, too, the dentist has rights he owes to himself. It will not do to indulge his patient in any position he may choose to assume. Many dentists toil to the great detriment of their work, their comfort, and their health, by allowing patients to loll about, double up, or turn about as fancy pleases them. We should insist on their assuming positions advantageous to us and our work. The mere position of the head is of great importance. Some chairs are made so wide that our patients, unconsciously, get away from us, and we are obliged to lean half over them to get at our work, or have frequently to bring them back within proper reach.

But, independently of the position of our patients, there is much to be observed in our own habits of position. Standing on one leg for an hour at a time is, of course, injurious. Standing in a stooped position for a whole day is suicide. Twisting our body into a corkscrew to see our work is nonsense. Habitually leaning hard against the arm of the chair is sure to bring on internal complaints. Having our mouth so near the mouth of the patient as to breathe his breath and the rottenness in his mouth, is of course deleterious.

The dentist should stand erect, in an easy, self-possessed and self-supporting position, as much as possible. The more props and supports he has, the more he wants, the more awkward will be his position, and the more unhealthful. Spinal complaints, consumption, vertigo, and many other complaints would thus be avoided. His chair should be so easily raised and lowered, and turned to either side, and back and forward, that he can bring his patient in any position to suit his work. The head, especially should be controllable, in aspect and position, without serious inconvenience to the patient.—"Extracts from Editorial in Items of Interest."

---

THE DENTAL MIRROR.—This is the title of a new dental journal edited and published in the city of New York. The first (July) number was issued June 20th. The standing of this journal may be judged from the fact that it is conducted by Dr. A. Ottolengui, and we presume he receives the assistance of the able progenitors of the enterprise, who, we are informed, include the brightest professional talent in New York. The "Dental Mirror" is to be published monthly at 63 West Fifty-fifth street. Send for a sample copy.

